# Influence of the Design Solutions of Extruder Screw Mixing Tip on Selected Properties of Wheat Bran-Polyethylene Biocomposite

**DOI:** 10.3390/polym11122120

**Published:** 2019-12-17

**Authors:** Emil Sasimowski, Łukasz Majewski, Marta Grochowicz

**Affiliations:** 1 Department of Technology and Polymer Processing, Mechanical Engineering Faculty, Lublin University of Technology, 20-618 Lublin, Poland; e.sasimowski@pollub.pl; 2 Department of Polymer Chemistry, Institute of Chemical Science, Faculty of Chemistry, Maria Curie-Sklodowska University, 20-614 Lublin, Poland; mgrochowicz@poczta.umcs.lublin.pl

**Keywords:** biocomposite, biofiller, mixing tip, lignocellulosic thermoplastic composite (LTC), wheat bran, polymer processing

## Abstract

The study investigated the impact of the extruder screw design solution—the intensive mixing tip used—on the course of the extrusion process and the properties of the obtained biocomposite extrudate. A lignocellulosic wheat bran biocomposite based on a low-density polyethylene matrix was extruded. Three mixing tips of the screw were used interchangeably: apineapple tip, a cut rings tip, and a Maddock tip. The experimental tests carried out included the production of an extrudate with a mass content of bran altered within the range from 10% to 50%. Processing properties such as the melt flow rate (MFR) and mass flow rate of the extruded biocomposite were determined. Selected physical, mechanical, and structural properties of the biocomposite extrudate obtained with the use of the three tested mixing tips at five bran contents were tested.

## 1. Introduction

Polymeric materials are gradually replacing and displacing metals, paper, glass, and other traditional construction materials from common applications due to their low weight, good strength properties, freedom of design, and the low cost of material and its processing [1]. The rapidly growing demand for inexpensive materials caused an increase in the production and consumption of polymeric materials, which resulted in the generation of significant amounts of plastic waste [1,2]. Improper management of this waste has led to considerable environmental pollution [1]. Due to the long period of natural decomposition of plastics, landfilling, which is the main method of waste disposal, is highly inefficient. Therefore, there is a growing interest in environmentally friendly materials, which are degradable in environmental conditions and have satisfactory mechanical properties. They are intended to replace or at least reduce the consumption of traditional petrochemical plastics. The development and production of biodegradable materials seems to be the cheapest and most effective method of effective waste management [1,3,4,5]. Examples of thermoplastic polymers meeting these requirements include: polyvinyl alcohol, polycaprolactone, and polylactide produced by means of synthesis, as well as cellulose, chitin, and starch obtained directly from natural sources. The main obstacle to the mass use of biopolymers is often the complicated and rigorous process of production and processing, which contributes to many times higher prices compared to petrochemical plastics [3,4,6,7]. This is why the main objective of the intensive work on environmentally friendly materials carried out over the last 20 years was to search for materials with a relatively short degradation time and a much lower price than biopolymers. The result of this work was the use of plant components in the processing of polymeric materials and the creation of biocomposites, which, as it has turned out, have beneficial utility properties and also economic and environmental advantages [8,9,10]. The worldwide trend of filling petrochemical polymers with cellulose-based and lignin-based materials, which are usually plant fragments being technological waste from the agriculture, food, wood and textile industries, is still observed today [3,11,12,13,14,15].

Any material containing lignin and cellulose is treated as a lignocellulosic material. Such materials include wood, wood agricultural crops, agricultural waste, and waste from the processing of agricultural products in the food or textile industry [16]. Lignocellulosic materials come in different forms because they are different parts of plants, and thus may have different chemical compositions [15,16]. The composition of lignocellulosic materials can be divided into three main components: cellulose, hemicellulose, and lignin. Thus, the properties of lignocellulosic materials, including their strength, are highly correlated with their chemical composition [16,17,18,19,20,21,22].

Thermoplastic polymers filled with all kinds of plant products and materials are referred to as lignocellulosic thermoplastic composites (LTCs) [23,24,25] in the matrix of both biodegradable and petrochemical polymers [23,26,27,28]. Numerous scientific studies have proved that organic materials based on cellulose and lignin are an appealing filler for petrochemical plastics. Significant studies on the use, application, and properties of LTCs containing inexpensive and renewable natural fillers have been carried out [6,29,30,31,32]. Natural materials of plant origin from annually renewable sources are now widely used as fillers for thermoplastic matrices, where they successfully replace synthetic fibres and fillers based on carbon, glass, and aramids as well as mineral fillers [8,33,34,35,36,37]. The literature includes studies on the use of different natural fillers constituting different parts of plants. Examples of such fillers include rice bran and husks [17,23,38], wheat bran [21], hard hazelnut shells [39], walnut shells [40], peanut shells [3], coconut shells [41], argan nut shells [42], dust from different tree species [15], by-products from palm oil production [43] and bagasse [24], date palm dust [16], red pepper stems [44], wheat straw [45], sunflower and maize stems [46], barley husks [47], and many others.

Many studies show that the main advantage gained from the use of natural fillers is the possibility of a high filling level, and consequently, a significant reduction in the consumption of petrochemical polymers and the induction of biodegradable properties, which limit the storage time of post-consumer waste [6,48]. Studies [4,5] have shown that a 50% addition of a natural filler causes a 15% weight loss observed during 9 weeks. Additional advantages of natural fillers are the lack of environmental toxicity, low cost, renewability, common occurrence, and low cost of manufacturing and processing, because they only require drying, grinding, and screening in order to separate fractions of desired dimensions before their introduction into the plastic [8,48,49,50]. Furthermore, the presence of natural fillers provides an attractive appearance and improves some physical properties by lowering the density, increasing the sound absorption coefficient [51], and improving the thermal insulation properties [14,52]. In addition to the benefits described above, the use of fillers made from natural waste reduces the negative impact of the storage and disposal of such waste on the environment [44,50,53]. Therefore, the use of lignocellulosic fillers constitutes a modern and progressive approach to the issues of waste management and processing of polymeric materials [14,54,55].

Despite the many benefits, the use of natural waste fillers presents some difficulties and limitations. Such features of materials containing lignocellulosic fillers as their strong hygroscopicity, absorption of large amounts of water, and the tendency to be inhabited by fungi may hinder or prevent their use in conditions of high humidity and exposure to microorganisms; but at the same time, these factors may accelerate their biodegradation via landfilling. They also make it necessary to dry the filler before processing [1,3,14,56]. Thermoplastic lignocellulosic composites in most cases have worse mechanical properties than the polymer matrix itself, which is caused by the lack of compatibility between the matrix and the filler surface [1,8,14,39,42,50]. This is due to the presence of hydroxyl groups, which give the filler surface hydrophilic properties, hinder its wettability by non-polar polymers during processing, and reduce the intensity of interactions at the interface [3,42,57,58]. To address this issue, chemical modifications of the filler are applied by mercerisation, benzoylation, acetylation, silanation, or grafted copolymerisation [59,60], which couple the hydrophilic filler with the hydrophobic matrix, forming indirect chemical bonds [61,62]. Nevertheless, many researchers have abandoned the use of compatibilizers, thus achieving only an improvement in stiffness, but at the same time a decrease in tensile strength and elongation at break, which are still often satisfactory [1,14,50,63]. This is economically justified, because the high price of the compatibilizer eliminates the benefits of using a less expensive natural filler. Another factor determining LTC properties is the quality of filler distribution in the polymer matrix, which has a decisive influence on the strength and elongation at break. In the case of most powder fillers, large particle size aggregates begin to appear at 20%–30% and start to obscure the differences between the different fillers. Very fine fillers are difficult to distribute in the polymer matrix, and the agglomerates formed behave as one large particle and constitute the place of crack initiation. Good dispersion and distribution can be achieved by effective mixing and an appropriate processing method [18,22,61,62,64]. The most commonly used method of polymeric composites processing, including those with natural fillers, is extrusion [65]. Extrusion produces both LTC finished products and composite granules, which are a starting material for subsequent processing [66]. The quality of polymer and filler mixing that occurs in the extruder plasticizing system, also known as mechanical homogenization, determines the specific features of the finished product, including its appearance, as well as its mechanical and optical properties. The mixing done in the extruder’s plasticizing system can be divided into shear mixing, also known as dispersive mixing, and distributive mixing. Distributive mixing can be further divided into longitudinal and transverse mixing. Longitudinal mixing is a result of the overlap of pressure and drag flows in the screw channels. The drag flow results from the impact of the flow rate component exerted perpendicularly to the screw line. The flow rate is the highest at the screw surface and equal to zero at the barrel surface. In contrast, the backward pressure flow is caused by an increase in pressure in the screw channel, and it is the opposite of the drag flow. Interaction between the above flows leads to an exchange of material between the layers due to differences in the dwell time of individual polymer particles in the plasticizing system. Transverse mixing consists of unifying the composition of a polymer/filler system in the section perpendicular to the extrusion direction. The reduction of difference in the polymer composition occurs as a result of the distribution of additional ingredients in the polymer due to the impact of shear stress during polymer flow in the plasticizing system. There is no exchange of material between individual layers. This is because the transverse flow, which is responsible for this type of mixing in the system, is caused by the impact of the polymer flow rate component perpendicular to the helix of the processing screw, and thus has no effect on the polymer mass flow rate. However, it causes polymer mixing and heating due to internal friction and plays an important role in polymer homogenization. When describing homogenization and polymer flow in the plasticizing system, one should also mention the phenomenon of leak flow, which does not occur in the screw channel but takes places between the internal surface of the barrel and the upper surface of the screw flight. Leak flow is caused by increased pressure in the screw channel and the impact of the polymer flow rate component perpendicular to the screw helix, but its effect on the extrusion process is negligibly small when compared to drag and backward pressure flows. The shear mixing in extrusion consists of overcoming cohesion forces of bigger material particles and their refinement due to the shear stress impact during polymer flow. The appropriate degree of homogeneity is achieved by the use of screws or replaceable screw parts with a specific geometry enabling distributive or dispersive mixing, depending on the type of filler used, in the metering section. Parts intended for intensive mixing of the processed material most often have rows of pins or spikes, whose purpose is to separate the flowing stream of plastic, or longitudinal edges parallel to the axis of the screw, grinding the flowing material on the internal surface of the cylinder [67,68]. 

The correct determination of processing conditions, including the geometric design solution of the screw, requires the knowledge of the mechanism of flow of highly filled polymeric materials, because the introduction of a natural filler into a polymer matrix significantly deteriorates its processability [69,70,71]. This is connected with a significant increase in viscosity together with an increase in the content of the filler [72,73], which is also affected by the size, shape, and structure of its particles [71]. The described changes in viscosity have a direct impact on the decrease in the flow rate of the plasticized composite material, and as a consequence, on the efficiency of the process as well as on the increase in the load of the extruder drive system [71].

On the basis of the reviewed literature on the production of biocomposites with a polyethylene matrix, it can be stated that there are few studies addressing the issue of the influence of structural solutions of the plasticizing system on biocomposite properties. Most of the available studies focus on changes of biocomposite properties caused by the chemical modification of their lignocellulosic fillers or the whole biocomposition. The issue regarding the influence of structural solutions of intensive shearing and mixing parts of the processing screw on the properties of the extruded polyethylene/bran biocomposite is almost absent from the literature. The set of applied processing conditions, selected by trial and error, specified by many authors does not even allow one to reproduce the experiment, as in their works, the authors focus mainly on the properties of the processed material. The study of the relationship between the design of the screw-mixing tip used, the processing characteristics, and the properties of the extrudate may be the basis for further research on the improvement of the properties and composition of LTC biocomposites. The proper selection of broadly understood processing conditions for new biocomposite materials often causes a number of difficulties, which may hinder and discourage further research and implementation of these materials in industry.

The aim of the study was to investigate the impact of the content of a lignocellulosic filler in the form of ground wheat bran and the design of the screw intensive mixing tip of a single-screw extruder on the efficiency of the extrusion process and selected physical, mechanical, and structural properties of the obtained lignocellulosic biocomposites based on a low-density polyethylene matrix.

## 2. Experimental

### 2.1. Test Stand

**Figure 1:**
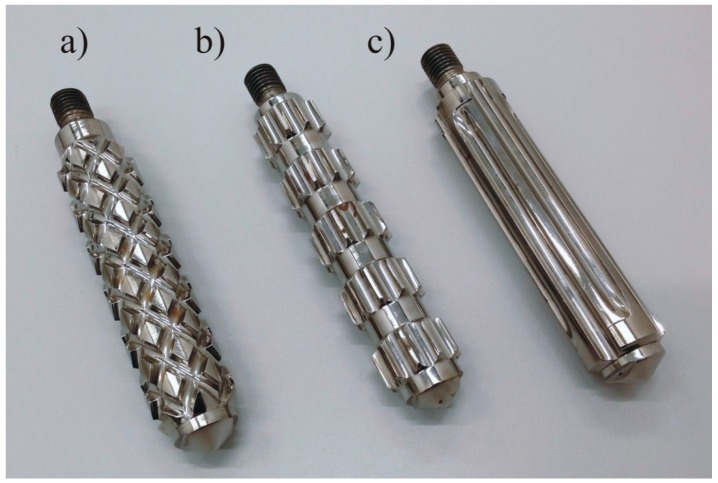
View of intensive mixing tips: (**a**) pineapple tip (**b**) cut rings tip, and (**c**) Maddock’s tip.

The extrusion process was carried out with the use of a single-screw extruder equipped with a three-zone processing screw with a diameter (*D*) = 32 mm and a ratio of the working part with a flight to the diameter *L*/*D* = 23, compression ratio (CR) = 12/5, with an exchangeable intensive mixing tip with a length *L*/*D* = 4.4. Three intensive mixing tips were used in the research: a pineapple tip, a cut rings tip and a Maddock tip, as shown in Figure 1. The pineapple tip and the cut rings tip are characterized by distributive mixing, while the Maddock tip was characterized by dispersive mixing, but some distributive mixing also takes place [74].

For extrusion, a rectangular section nozzle was used to produce a 25 mm × 2.5 mm strip. The extrudate was cooled in a cooling tank.

### 2.2. Research Program and Methodology

The experimental tests were conducted in accordance with a complete plan, in which the following variable factors were assumed: the pineapple, Maddock, and cut rings mixing tips, as well as the mass content of wheat bran of 10%, 20%, 30%, 40%, and 50%. Measurements were carried out in five repetitions.

**Figure 2:**
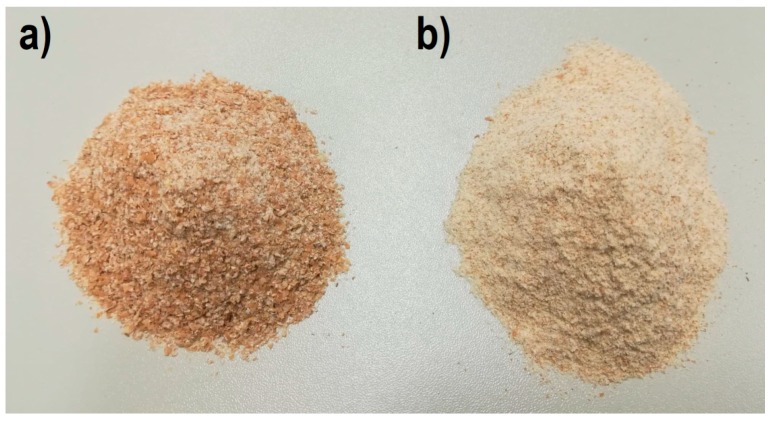
View of wheat bran powder: (**a**) immediately after grinding, and (**b**) after separation of fractions with a grain size of less than 0.4 mm.

Dowlex 2631.10EU low-density polyethylene produced in powder form by Dow Chemical Company [75] and filled with wheat bran (WB) from a local mill was extruded. Wheat bran is technological waste from the production of white flour and takes the form of thin flakes of up to a few millimetres in size. Bran preparation included grinding with a grain mill (Figure 2a), drying in a heating chamber for 24 h at a temperature of 60 °C, and then screening on a sieve tray column to obtain fractions with a particle size of less than 0.4 mm (Figure 2b). After weighing, the components of the composite were premixed using a gate mixer.

The extrusion process was carried out at a constant screw speed of 50 rpm. The temperature in the individual heating zones of the plasticizing system was 105, 110, 120, and 135 °C, and the temperature of the extruder head was 125 °C. The extruder was fed by gravity, and during the extrusion process, a constant level of the mixture was maintained in the feed throat of the extruder.

The experimental tests carried out included the following:

- Observation of the structure of the extrudate samples using a NIKON optical microscope model Eclipse LV100ND equipped with a DS-U3 camera. Image analysis was performed in the NIS-Elements AR 4.20.00 software. The extrudate samples were prepared for microscopic observation by making their fractures perpendicular to the direction of the extrusion. In order to achieve a brittle breakthrough, the strips were cooled in liquid nitrogen for one minute before breaking. The dark field method was used for observation;
- Normal density measurement of the extrudate ρ g/cm^3^, in accordance with EN ISO 1183-1:2019 [76], using a Radwag AS 82/220.R2 digital scale with a density measurement attachment;
- Measurement of water absorption in percentage. In order to determine the water absorption, the obtained extrudate was predried in a thermal chamber and weighed. Then, the samples were kept in water at 22 °C for 7 days. Then, the extrudate samples were weighed again, and water absorption was determined in accordance with EN ISO 62:2008 [77];
- Measurement of the moisture content of the extrudate in accordance with EN ISO 585:1990 [78] with a Radwag WPS 50SX moisture analyzer. The obtained extrudate was predried in a thermal chamber at 40 °C for 24 h. Then, the samples were stored in a room with a humidity of 25% at 22 °C. The moisture content of the extrudate after drying, after 24 h, and after 7 days was measured at 105 °C with the use of a moisture analyzer;
- Determination of the tensile strength of the extrudate σ [MPa], relative nominal elongation at maximum tensile stress ε [%], and Young modulus [MPa], using a Zwick/RoellZ010 test machine in accordance with EN ISO 527-2 [79] at a tensile speed of 50 mm/min;
- Color measurement of the extrudate samples using an X-Rite Ci4200 spectrophotometer. The color was described in the CIELab system, where it is defined in a L*, a*, b* space. The a parameter describes the color from green (negative values) to red (positive values), the b parameter describes the color from blue (negative values) to yellow (positive values), while the *L* parameter is the luminance–brightness, representing the gray scale from black to white (value 0 corresponds to black and value 100 corresponds to white). The difference between two colors—two points in the three-dimensional lab space—can be expressed with the following function:
  $$\Delta E=\sqrt{\Delta L^{2}+\Delta a^{2}+\Delta b^{2}}$$
  whereΔL, Δa, Δb are the difference in color parameters between the samples being compared, respectively;
- Melt flow rate index (MFR) (150 °C/5 kg) [g/10 min]. The measurement was performed with a CEAST extrusion plastometer model MeltFlow TQ6841 based on the recommendations of EN ISO 1133-1:2011 method A [80].
- The mass flow rate of the extruded plastic material G g/s; the extrudate samples were taken at constant time intervals and then weighed using a Fawag TP-3/1 scale. Flow rate was calculated by dividing the mass of the extrudate by the extrusion time;
- AnFTIR analysis of the samples was performed using an FTIR TENSOR 27 spectrophotometer (Bruker, Germany) equipped with an ATR attachment with a diamond crystal. Spectra (16 scans per spectrum) were collected in the range of 600–4000 cm^−1^ with a resolution of 4 cm^−1^;
- DSC tests of the obtained extrudate were performed using a DSC 204 F1 Phoenix differential scanning calorimeter (NETZSCH, Günzbung, Germany) and NETZSCH Proteus data processing software. Crystallinity degree *X_c_* and melting enthalpy Δ*H* were determined, along with the melting point *T_m_* and crystallization temperature *T_c_* of the obtained extrudate. The crystallinity degree was calculated using the following function:
  $$X_{c}=\left(\frac{\Delta H}{\left(1-u\right)\times \Delta H_{100\%}}\right)\times 100\%$$
  assuming that for PE Δ*H*_100%_ = 293 J/g [81]. DSC curves were recorded in the system for heating(I) from −140 to 150 °C (10 K/min), for cooling from 150 to −140 °C (10 K/min), and for heating (II) from −140 to 150 °C (10 K/min). The analyses were carried out in an inert gas (argon) atmosphere at a gas flow rate of 20 mL/min. The samples were analyzed in aluminium crucibles with a pierced lid;
- TG tests of the extrudate in oxidizing atmosphere were carried out using an STA 449 F1 Jupiter thermal analyzer (NETZSCH, Günzbung, Germany) combined with the FTIR TENSOR 27 spectrophotometer (Bruker, Germany), which enabled the simultaneous analysis of gaseous products of the decomposition of the analyzed samples. The analyses were carried out at 40–900 °C in synthetic air, at a flow rate of 25 mL/min. Samples weighing about 12 mg were analyzed in crucibles made of Al_2_O_3_.

Microscopic observations of the structure were carried out for selected samples of the obtained extrudate. Samples with the lowest (10%) and the highest (50%) bran content obtained with the use of the three screw mixing tips were examined. 

## 3. Results

The results of the experimental tests were statistically elaborated in STATISTICA 13 software using analysis of variance (ANOVA). At the beginning, the required assumptions, such as the normality of variable distribution (the Shapiro–Wilk test), and the homogeneity of variance (the Levene’s or the Brown–Forsythe tests) were checked. The Welch’s *t*-test was used wherever a heterogeneity of variance was observed. The Tukey’s multiple comparisons test was conducted once the analysis showed that statistically significant differences were visible between the examined average values. The test, also called a posthoc test, enables the grouping of average values and the separation of homogeneous groups that are not statistically significantly different from each other. The analyses were conducted at the level of significance *p* = 0.05. The obtained results were plotted on graphs with average values, and 95% confidence intervals marked.

### 3.1. Microstructure

**Figure 3:**
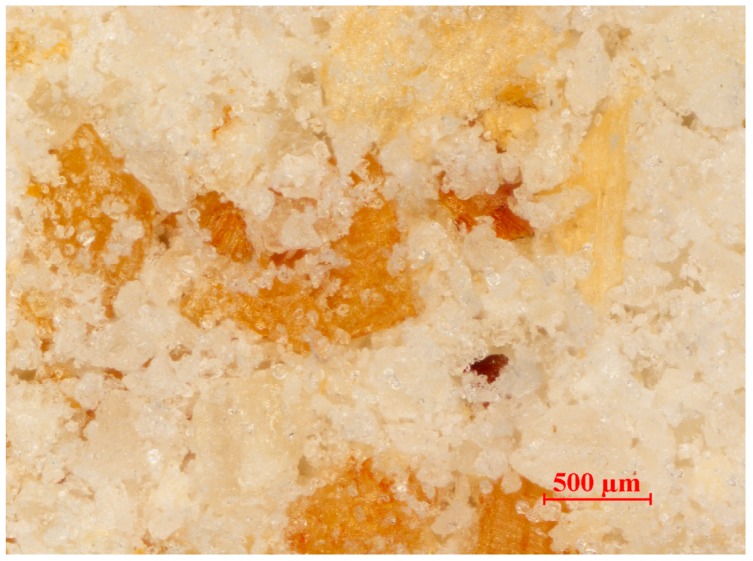
Microscopic image of wheat bran powder, fractions with a grain size less than 0.4 mm.

Figure 3 presents a microphotograph of the filler used, which was prepared from ground wheat bran with a grain size less than 0.4 mm. In terms of quantity, white crystals with dimensions much smaller than the accepted upper limit of 0.4 mm and shape similar to a sphere are clearly dominant. Among them, there are several times larger thin flakes of brown or yellow color, the length and width of which are close to the maximum adopted dimension, while the thickness is clearly smaller than the other two dimensions.

**Figure 4:**
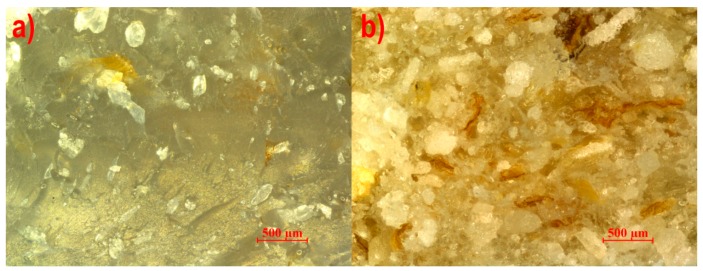
View of the breakthrough of the extrudate with a bran content of (**a**) 10% and (**b**) 50% made using the pineapple mixing tip.

**Figure 5:**
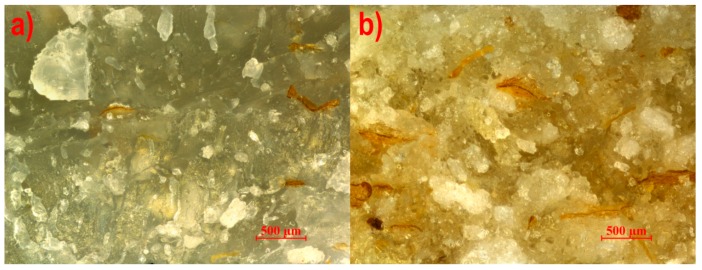
View of the breakthrough of the extrudate with a bran content of (**a**) 10% and (**b**) 50% made using the Maddock mixing tip.

**Figure 6:**
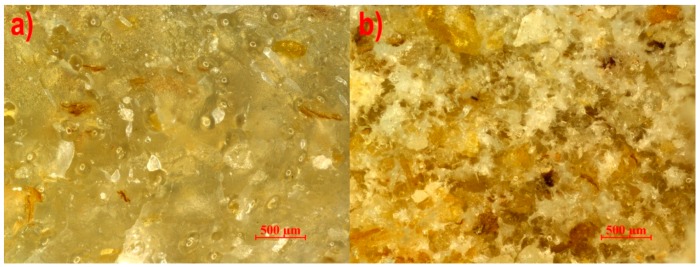
View of the breakthrough of the extrudate with a bran content of (**a**) 10% and (**b**) 50% made using the cut rings mixing tip.

The microstructures of the studied composites extruded with the pineapple tip, the Maddock tip, and the cut rings tip are presented in Figure 4, Figure 5 and Figure 6, respectively. Extreme content was selected for analysis to reveal the tendency of changes in the microstructure caused by the increasing content. It is plainly visible that in all the photographs, thin brown-colored flakes have significantly smaller linear dimensions than in photo 3, which may mean that these filler particles have little strength and are crushed by the screw during processing. No privileged orientation of the flakes was found. White crystals surrounded by a thin layer of plastic were visible on the surface of breakthroughs. Pores were observed for all three studied screw tips, which may be a result of water vapor emission from bran particles. However, for the cut rings tip, pores were noticeably larger and occurred in a much greater number than for the other two tips. The reason for this can be found in the geometry of the cut rings tip, where the volume of the channel filled with the plasticized material is greater than in the case of the pineapple tip and the Maddock tip, which results in a lower material pressure for this tip, allowing the formation of pores with a larger volume. By comparing photographs of samples with 50% bran content, it may be concluded that bran particles have a better adhesion to polyethylene when the pineapple and Maddock’s tips are used than when the cut rings tip is in use. Photos 4b and 5b show white crystals surrounded by a plastic layer on the surface of the breakthrough, which means that the breakthrough line bypassed the filler particles. On the other hand, only a few white crystals are visible in Figure 6b; however, flocky structures appeared, which were a remnant of the extraction of bran particles from the layers of polyethylene covering them, forming the walls of pores.

### 3.2. Density

**Figure 7:**
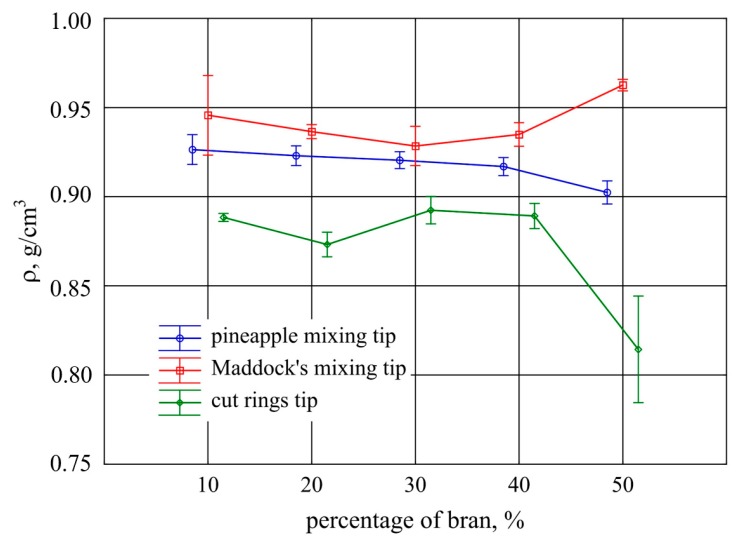
Density of the extrudate obtained with the screw mixing tips tested, depending on bran content (mean values with 95% confidence intervals).

Figure 7 illustrates the determined density values of a normal extrudate produced with different bran contents using the three screw mixing tips. The statistical analysis of the results (Welch’s *t*-test) showed that there were statistically significant differences between them. The Tukey’s posthoc test showed that in the case of the compared mixing tips, increasing the content of bran did not influence the change in density, and statistically significant differences were observed only at the highest content of 50%. The Maddock tip increased density, while the pineapple tip and cut rings tip decreased density. The highest density was discovered for the extrudate obtained using the Maddock and the pineapple mixing tips. In the range of 10%–40% bran content, the density of the extrudate obtained with the use of both these tips was similar. Significantly lower density values of the extrudates were obtained for the cut rings mixing tip. This was probably due to the large number of air bubbles that can be seen on the microscopic images of the breakthroughs of the extrudate obtained using this mixing tip. The densities obtained at 30% and 40% bran content were comparable to those obtained at 50% bran content with the use of the pineapple tip.

It should be emphasized that the density values determined in line with the standard were not as predicted. The density of bran determined using a pycnometric method (ISO 1183-1:2019) was 1.472 g/cm^3^, and the obtained densities of the tested compositions were lower than the density of water and comparable to the density of polyethylene, which was 0.935 g/cm^3^. The probable cause is the presence of bubbles visible in microscopic images, which increased the volume of the extrudate.

### 3.3. Water Absorption

**Figure 8:**
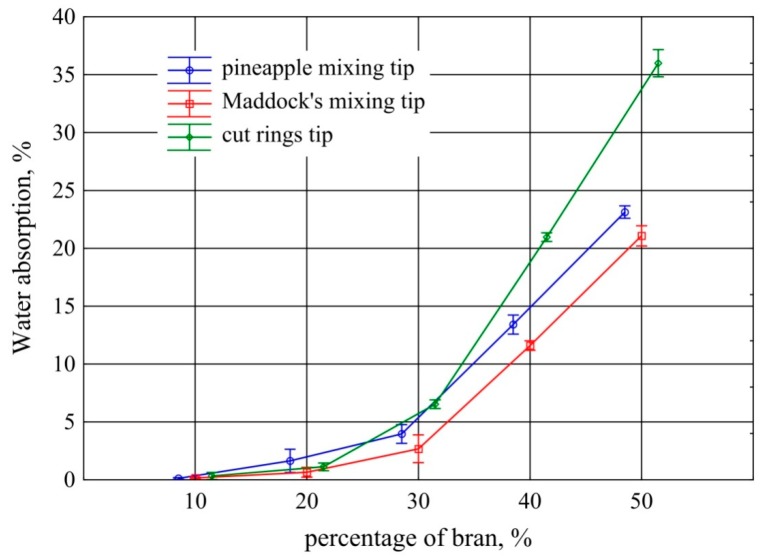
Water absorption of the extrudate obtained with the screw mixing tips tested, depending on bran content (mean values with 95% confidence intervals).

The results of measurements of the water absorption of the extrudate obtained using the screw mixing tips that were tested, depending on bran content, are shown in Figure 8. The analysis showed that statistically significant differences in water absorption between the used mixing tips of the screw begin to occur at 30% bran content. Significantly higher water absorption is characteristic of the extrudate obtained with the use of the cut rings tip. At 40% bran content, water absorption was the same as that of the extrudate with 50% bran content obtained with the Maddock tip. This is due to the penetration of water into numerous bubbles of considerable size present in this extrudate. In addition, similarly to moisture absorption, the lowest water absorption is characteristic of the extrudate obtained with the use of the Maddock tip.

On the basis of the obtained water absorption changes, it can be concluded that the plasticized material effectively covers filler particles with a thin layer during processing up to 30% of bran content, limiting water absorption only to the surface layer [23,27]. There, water is absorbed directly by the exposed bran fragments and cannot penetrate into the extrudate through the plastic layers. With 40% of bran, there is a steep increase in water absorption, which means that the plastic no longer fully covers filler particles that come into contact with each other and allow water to penetrate into the extrudate. For comparison, the measurements showed that polyethylene did not exhibit the ability to absorb water.

The water absorption test also indicated the reason for obtaining low density values, even at high bran contents. All the samples with bran content ranging from 10% to 30% floated on the water surface, while the samples with 40% and 50% dropped to the bottom of the vessel. However, the descent took place only after about 2 days. This means that the process of soaking water into the sample is a lengthy one, and a short-term immersion in water during a density test does not allow the filling of hollow spaces inside the bubbles. Hence, low values were obtained in the density test, even at high filler contents.

### 3.4. Moisture Content

**Figure 9:**
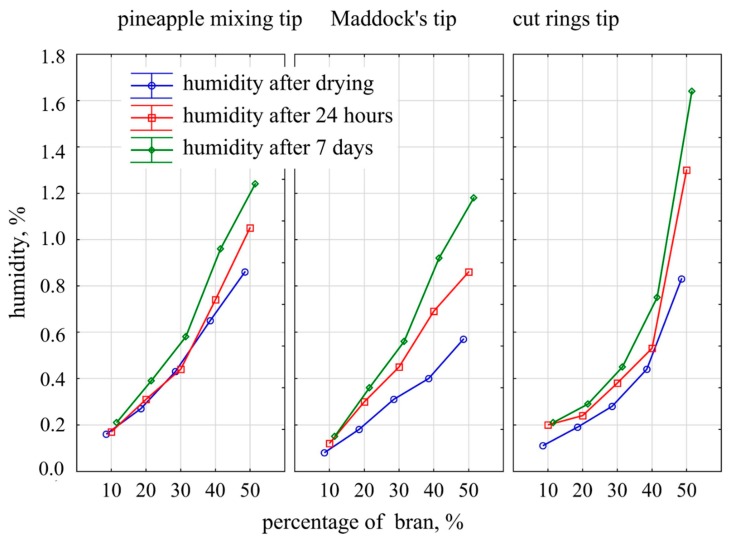
Moisture content of the extrudate obtained with the use of the screw mixing tips tested depending on the time and bran content.

The obtained results of moisture content of the samples, which was measured directly after drying and then after 24 h and after 7 days of storage in a room with 25% air humidity and 22 °C temperature, are presented in Figure 9. It was observed that with the increase in the content of bran, the moisture content of the extrudate increased significantly. In the case of the pineapple and Maddock mixing tips, this was a proportional increase, whereas in the case of the cut rings tips, it was exponential. The moisture content of the extrudate obtained with the pineapple tip measured after drying had a higher gain of 0.18% moisture per a 10% increase in bran content than that of the extrudate obtained using the Maddock tip, which exhibited a 0.13% gain. The extrudate moisture measured after 24 h for the pineapple tip was also slightly higher than that of the Maddock tip extrudate. However, after 7 days, the moisture content of the extrudate obtained with the use of these two mixing tips was comparable, and had risen by 0.26% per a 10% increase in the content of bran. The extrudate obtained by using the cut rings tip in a range of up to 40% of bran content had a similar, and sometimes even lower, moisture content compared to that obtained by using the other tips. Still, for the bran content of 50%, the moisture measured after 24 h and 7 days was much higher, by 0.25% and 0.4% respectively, compared to the pineapple tip. It can be assumed that it was the effect of moisture penetration into deeper layers of the extrudate, which was caused by the less effective covering of filler particles by the material shown in the microscopic photographs.

For comparison purposes, samples of extrudate made of polyethylene alone without bran were also examined. The moisture content immediately after drying and after 24 h was 0.02% and 0.04% after 7 days.

### 3.5. Tensile Testing

**Figure 10:**
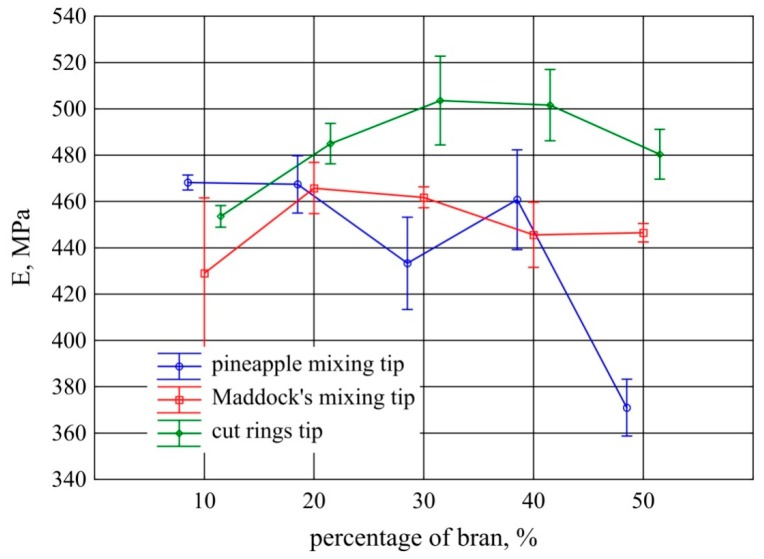
Young modulus of the extrudate obtained with the screw mixing tips tested, depending on bran content (mean values with 95% confidence intervals).

The obtained results of Young modulus measurements for the tested extrudates are presented in Figure 10. Statistical analysis showed that in the case of the Maddock tip, there were no significant changes in the modulus as bran content rose. When the pineapple screw tip is used, increasing bran content up to 40% also has no significant effect on the Young modulus. At the content of 50%, a significant decrease in the modulus was observed, and the average value of 371 MPa—the lowest value among the tested extrudate samples—was obtained. A statistically significant increase in the modulus was observed in the case of the cut rings tip. It occurred in the range of 30%–40% of bran content. At other contents, the results obtained for the extrudate produced with this tip did not differ significantly. 

The Young modulus of extrudate samples made from polyethylene alone without bran was 426 MPa with a standard deviation of 37.3 MPa.

**Figure 11:**
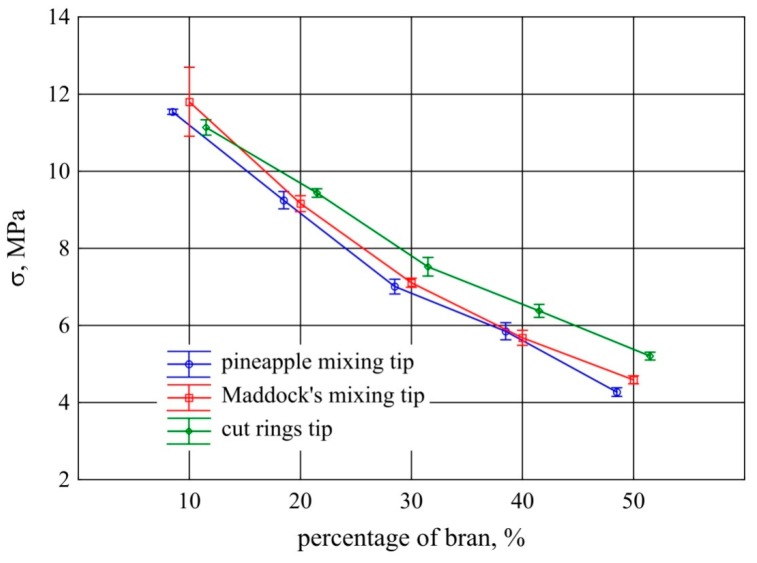
Tensile strength of the extrudate obtained with the screw mixing tips tested, depending on bran content (mean values with 95% confidence intervals).

**Figure 12:**
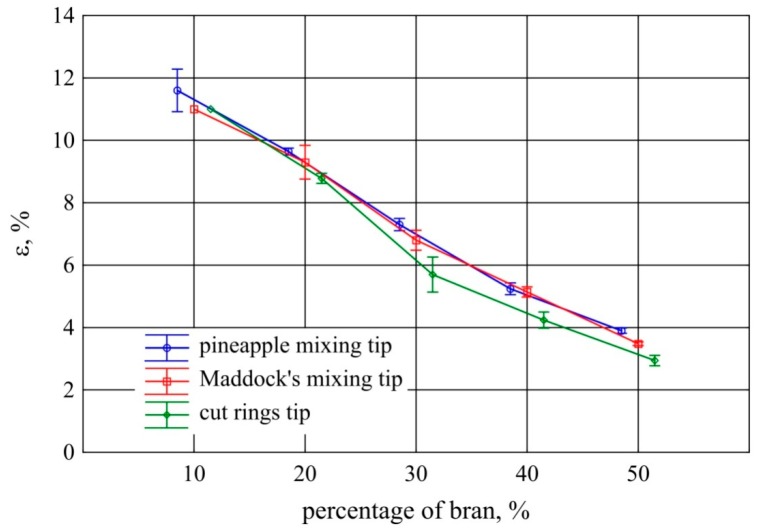
Strain at tensile strength of the extrudate obtained with the screw mixing tips tested, depending on bran content (mean values with 95% confidence intervals).

Tensile testing of the extrudate samples showed a great impact of both bran content and screw tips on tensile strength. This was confirmed by the statistical analysis carried out. The tensile strength and the corresponding deformation of the samples decreased proportionally with the increase in bran content (Figure 11 and Figure 12). When using the pineapple and Maddock tips with an increase in the content of bran by 10%, tensile strength decreased on average by 1.76 MPa and strain by 2%. The results obtained with the use of these two tips for each of the examined bran contents did not differ significantly. On the other hand, the tensile strength of extrudate samples obtained using the cut rings tip was considerably higher in the range of 30%–50% of bran content. This is also reflected in the results of strain at tensile strength. It was clearly lower for the cut rings tip in this range of bran content than for the other screw tips tested (Figure 12). In the case of the cut rings screw tip, the proportional decrease in strength along with the gain in bran content was also smaller and amounted to 1.48 MPa per 10% increase in bran content. Similar courses of changes in tensile strength properties were obtained by the authors of works [14,42,53] on the application of lignocellulosic fillers.

The tensile strength of the polyethylene extrudate samples without bran was much higher and amounted to 21.8 MPa, with a standard deviation of 1.3 MPa. On the other hand, strain at tensile strength was an order of magnitude higher and amounted to 737% on average, with a standard deviation of 32%.

### 3.6. Color

**Figure 13:**
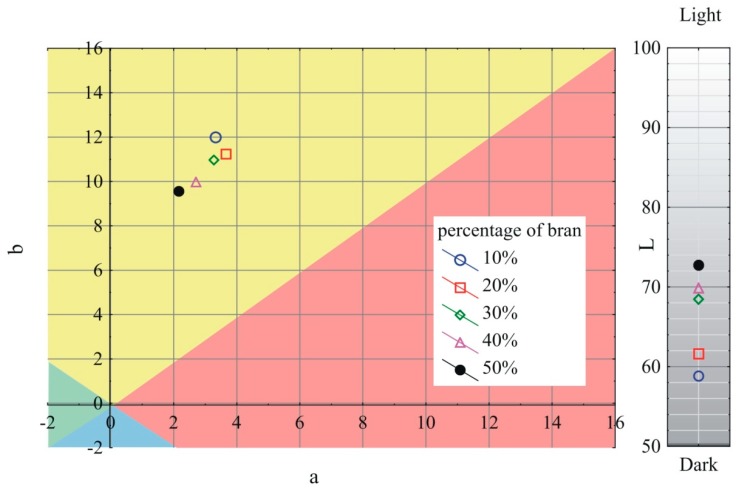
Variability of the L, a, and b color parameters of the extrudate produced using the pineapple mixing tip, depending on bran content.

**Figure 14:**
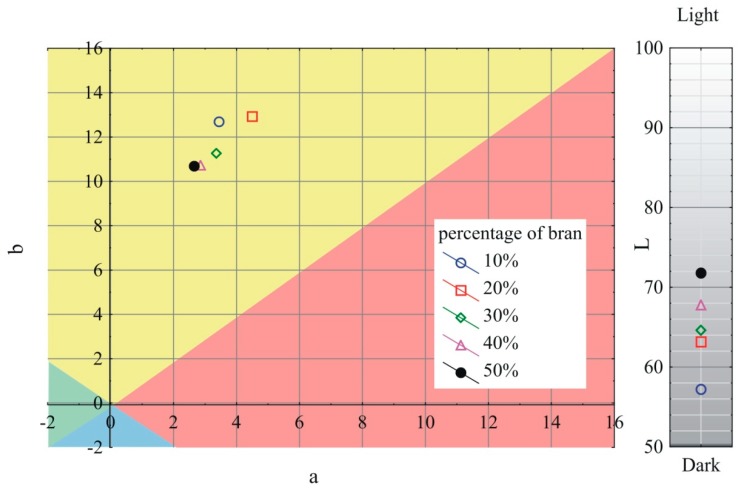
Variability of the L, a, and b color parameters of the extrudate produced using the Maddock mixing tip, depending on bran content.

**Figure 15:**
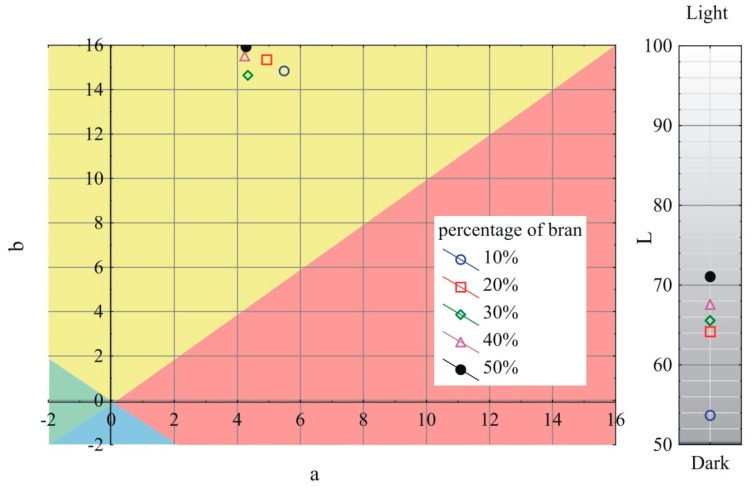
Variability of the L, a, and b color parameters of the extrudate produced using the cut rings mixing tip, depending on bran content.

The obtained results of the measurements of the *L*, a, and b color parameters variability of the extrudate produced using the screw mixing tips tested, depending on bran content, are shown in Figure 13, Figure 14 and Figure 15. It has been observed that with increasing bran content, the color of the extrudate obtained using all the screw tips studied gradually changes, starting from the mean differences of 2 < ΔE < 3.5 recognizable by an inexperienced person at bran content up to 30%, through clear color differences of 3.5 < ΔE < 5 for 40% bran content, up to large color differences of ΔE > 5 at 50% bran content. Increasing the content of bran increases the luminance of the obtained extrudate; it becomes whiter/lighter, which is connected with a noticeable content of bright particles visible in microscopic photographs. At the same time, the a parameter is reduced, and the color of the extrudate becomes greener. The b parameter in the case of the pineapple and Maddock screw mixing tips decreases as the bran content rises and the color of the extrudate becomes bluer. However, when the cut rings tip is used, the b parameter is slightly increased, and the color of the extrudate leans toward yellow. It should be noted that the color of the extrudate obtained by using the cut rings tip was different from that obtained by using the other screw tips. Its color was darker and shifted toward yellow and red. This may indicate a different mechanism of filler particles distribution in the cross-section of the extrudate and a tendency to locate darker flakes, which were visible in Figure 3, closer to the extrudate surface. The registered values of parameters L, a, and b describing the color of unfilled polyethylene were 67.3, −0.55, and −3.01 respectively.

### 3.7. Melt Flow Rate

**Figure 16:**
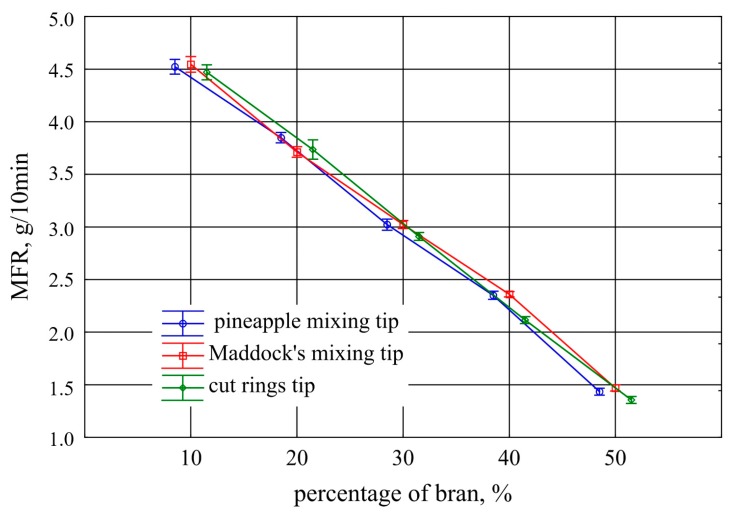
Melt flow rate (150 °C; 5 kg) of the extrudate obtained with the screw mixing tips tested, depending on bran content (mean values with 95% confidence intervals).

Melt flow rate (MFR) tests conducted on the extrudate samples are shown in Figure 16. A very clear proportional decrease in MFR value was observed, on average by 0.77 g/10 min per 10% increase in bran content. Statistical analysis also showed that the results obtained for the extrudates produced with the Maddock tip and the pineapple tip were comparable, and were slightly lower for the cut rings tip extrudate from 30% bran content. This was due to the similar shear rate of the two tips for the processed plastic and a lower shear rate of the cut rings tip. On the other hand, the decrease in the melt flow rate index in the function of the filler content is an obvious effect of the composition viscosity increase caused by the presence of fine particles dispersed in the polymer matrix [72,73]. The determined melt flow rate index for polyethylene without wheat bran was 5.25 g/10 min with a standard deviation of 0.05 g/10 min.

### 3.8. Mass Flow Rate

**Figure 17:**
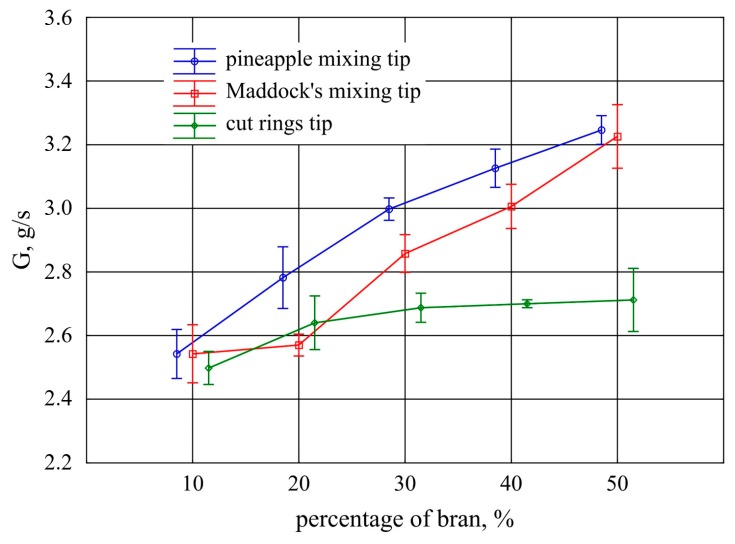
Mass flow rate of the extruded plastic obtained with the screw mixing tips tested, depending on bran content (mean values with 95% confidence intervals).

The impact of bran content on the mass flow rate of the extruded plastic using the tested screw mixing tips at constant screw speed is presented in Figure 17. The conducted analysis showed that statistically significant differences in mass flow rate between the tips used begin to occur from 20% of bran content. A significant gain in the plastic mass flow rate was observed along with the increasing bran content when the pineapple and Maddock mixing tips were used. As a result of the increase in the content of bran from 10% to 50%, an increase in the flow rate of 0.7 g/s on average was observed for both tips, which corresponds to an increase of 27%. The obtained results for both these tips do not differ significantly except for minor changes in the range of 20%–30% of bran content. In the case of the cut rings tip, a slight increase in the flow rate of 0.2 g/s (8%) on average was observed as a result of an increase in the content of bran above 10%. A further increase of bran content in the case of this mixing tip did not cause any statistically significant changes in the plastic flow rate. The reason was probably lower extrusion pressure arising from a higher volume of the channel in the cut rings tip, which can be confirmed by a more intensive growth of pores observed in Figure 6. The mass flow rate value obtained for polyethylene alone was 2.38 g/s with a standard deviation of 0.09 g/s.

### 3.9. FTIR Spectroscopy

**Figure 18:**
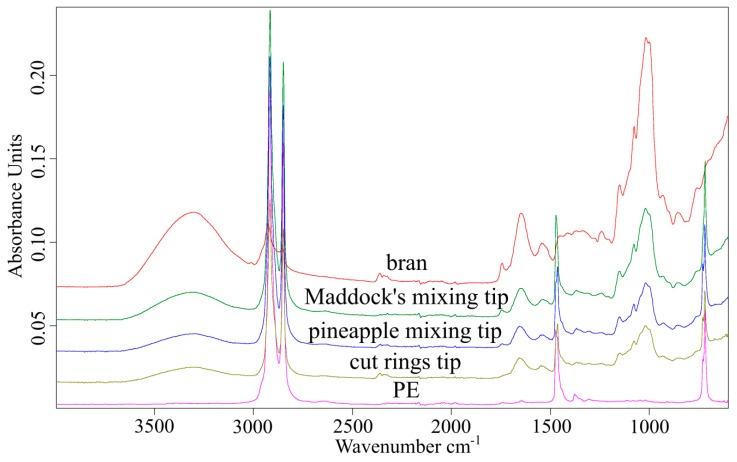
FTIR spectra of polyethylene (PE), wheat bran (WB), and composites containing 50% of wheat bran made using the Maddock (PE-M), pineapple (PE-P), and cut rings (PE-C) tips.

In order to check whether the extrusion process affects the changes in the chemical structure of bran and polyethylene, ATR-FTIR analysis was performed. The FTIR spectrum of polyethylene (Figure 18) shows characteristic absorption bands at 2916, 2849, 1471, and 1377 cm^−1^ from stretching and deformation vibrations of methylene groups. Additionally, the presence of an absorption band at 1377 cm^−1^ indicates the presence of branches in the linear structure of PE. On the spectrum obtained for the bran sample (Figure 18), characteristic absorption bands are observed for the following vibrations: 3311 cm^−1^ for vibrations of –OH groups (present in polysaccharides, phenolic compounds, and water); 2924 and 2854 cm^−1^ for vibrations of methyl and methylene groups; 1743 cm^−1^ for vibrations of C=O groups present in carbonyl compounds (in bran present mainly in pectin and hemicellulose) [82,83,84]; 1648 cm^−1^ for vibrations of the –OH group from water absorbed by starch [85,86] and vibrations of the amide group C–N present in proteins; 1546 cm^−1^ for vibrations of the N–H amide group present in proteins [87]; and lastly, 1150, 1078, 1017,and 999 cm^−1^ for vibrations of the C–O–C and C–O groups present in the structure of polysaccharides, especially starch, cellulose, hemicellulose, pectin, and lignin. The FTIR spectra of polyethylene(PE) and bran composite samples (Figure 18) show absorption bands present in the spectra of both components. No changes in absorption bands caused by the bioorganic component were observed, which indicates that no changes in its chemical structure occurred during the process of composite production.

### 3.10. Thermal Properties

**Figure 19:**
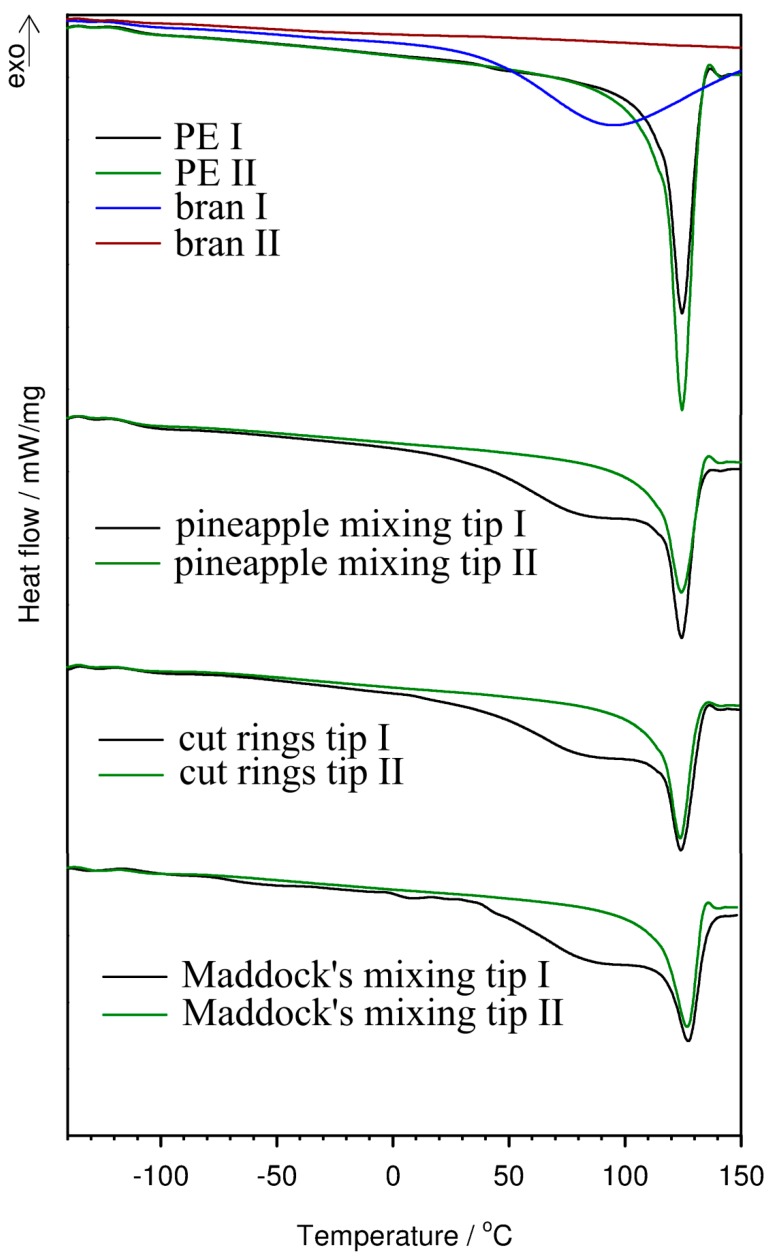
DSC curves of components and composites with 50% bran content obtained in an inert atmosphere.

The characteristics of crystallinity degree, melting point, crystallization temperature, and glass transition temperature of the extrudate were investigated by means of differential scanning calorimetry. The analysis of Table 1 data obtained from DSC curves (Figure 19) indicates that both the addition of a biocomponent and extrusion conditions narrowly impact the glass transition, melting, and crystallization temperatures of the extrudate as compared to pure polyethylene. However, a slight decrease in crystallization temperature and an increase in melting temperature may suggest that the structure of the extrudate contained larger crystallites of PE than those in pure PE. The crystallinity degree of the extrudate visibly decreased in comparison with pure polyethylene [88], especially for the extrudate obtained with the Maddock tip. Such a large addition of a biofiller, which due to its hydrophilic nature is not fully compatible with hydrophobic PE, causes the separation of polymer chains, reduces their mobility, and hinders the crystallization of PE. Similar behavior has been described for bioorganic composites containing lignocellulosic material and starch [1,89]. In the DSC curves of the first heating process, another additional endothermic effect is visible in the temperature range from 30 to 105 °C. It is caused by the evaporation of moisture contained in the extrudates. Since this effect occurs simultaneously with the process of polyethylene fraction melting, it was not possible to determine the degree of crystallinity of the extrudates in the first heating process. 

**Figure 20:**
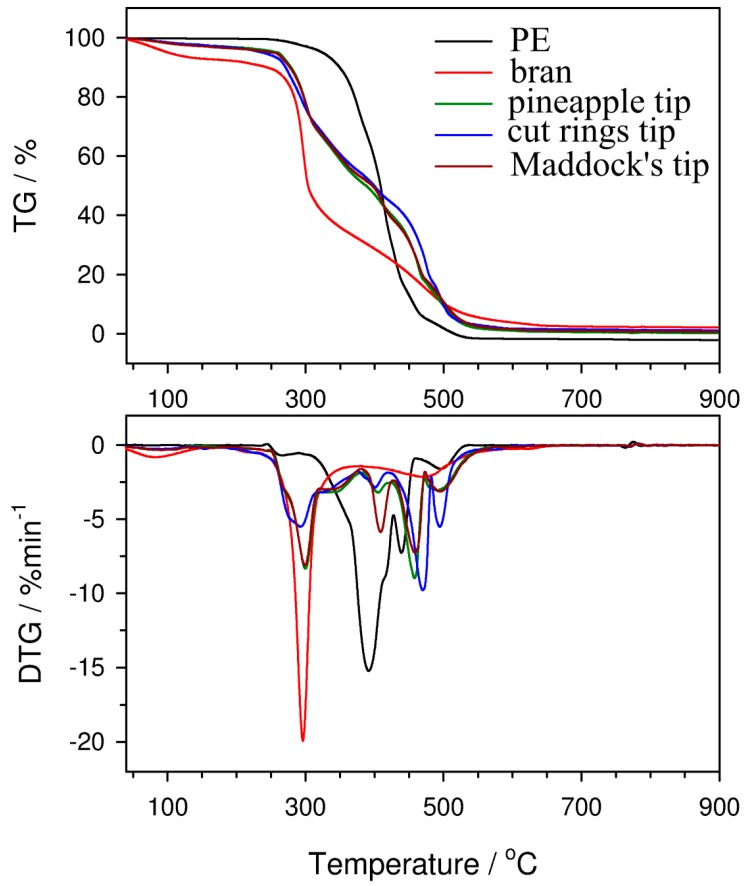
TG and DTG curves obtained in oxidizing atmosphere for polyethylene (PE), wheat bran, and composites containing 50% of wheat bran made using the Maddock, pineapple, and cut rings tips.

**Figure 21:**
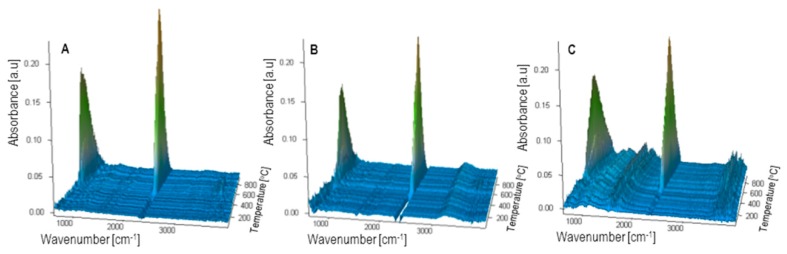
3D FTIR spectra of gaseous decomposition products; (**A**)—PE samples, (**B**)—composite containing 50% of wheat bran extruded with the pineapple tip, (**C**)—bran samples.

Knowledge of thermal resistance is necessary to determine the temperature of processing and the temperature of application of polymeric materials, including their bran composites. The process of composite manufacturing often requires the use of temperatures close to the decomposition temperature of the bioorganic component; therefore, the characteristics of its thermal resistance are important. Thermogravimetric measurements in an oxidizing atmosphere were performed in order to obtain the full thermal characteristics of components and moldings containing 50% of bran. Based on TG curves (Figure 20), the thermal resistance of the composites is lower than that of PE. The initial decomposition temperature (IDT, marked as 5% of the mass loss) for PE is 327 °C, whereas for the cut rings tip, it decreased to 236 °C (Table 2). The decline in thermal resistance is due to the presence of a filler that has a lower IDT value (180 °C). Moreover, in the case of the cut rings tip sample, the decrease in IDT is probably the greatest because bran particles were not fully covered with a polymer, as shown by microscopic tests. Pure polyethylene undergoes thermal degradation in three stages, with the maximum rate of mass loss at temperatures of 398, 445, and 502 °C. The decomposition is based on thermo-oxidation processes and its main product is carbon dioxide, whose emission is reflected by absorption bands at 2300–2365 and 669 cm^−1^ present in the 3D FTIR spectrum (Figure 21). The bran sample decomposes by oxidation in a two-stage process, which can be seen as two maxima in the DTG curve (Figure 20). The first stage with maximum decomposition at 296 °C is related to the oxidation of polysaccharides and proteins. This process takes place at similar temperatures for starch, proteins, and lignocellulose; therefore, no separate DTG peaks for individual bran components are observed under the conditions of the analysis. The second stage with maximum decomposition at 473 °C is related to the oxidation of the carbon residue produced after the first stage. Additionally, an 8% weight loss observed already at temperatures below 100 °C is caused by the emission of water absorbed in the sample. The susceptibility of bran to water adsorption results from the properties of the polysaccharides composing it, especially starch (the presence of which was confirmed by an FTIR analysis and a quality test with iodine) [85,86]. The obtained composite extrudates undergo thermal decomposition in five stages with the maximum rate of mass loss at temperatures of about 300, 339, 405, 458, and 491 °C. The first stage of decomposition is preceded by weight loss, reaching approximately 4.5% for all examined extrudate samples, caused by water desorption. In the first two stages of decomposition, the bioorganic fraction is oxidized; the next two stages of decomposition are related to the oxidation of the PE fraction, while the fifth stage is related to the oxidation of the produced residues. Surprisingly, an additional not fully separated peak at about 339 °C is observed on DTG curves. Its presence may be associated with the slowdown of bran decomposition due to its particles being covered with thermally more resistant polyethylene. This results in a slower diffusion of heat into the interior of the extrudate, and so the starch, protein substances, hemicellulose, and cellulose are first broken down (at about 300 °C). However, lignin, which is a cross-linked polymer containing aromatic groups and has a higher thermal resistance, decomposes at about 339 °C in the next stage. In the 3D FTIR spectrum of gases emitted during the decomposition of the extrudates (Figure 21), only absorption bands from carbon dioxide and water are observed, and the decomposition of composites takes place in a wider temperature range in comparison with individual components. This is caused on the one hand by the slower diffusion of heat into the sample, especially at higher temperatures, and on the other hand by hindered diffusion from the sample interior of the produced gaseous decomposition products [90].

## 4. Conclusions

The conducted tests confirmed that it is possible to use ground wheat bran as a filler of thermoplastics, even at a high filling level. 

Bran content in the biocomposite has the greatest impact on almost all the examined values. The results of the experimental tests showed that the design of the screw mixing tips also has a significant influence on the characteristics of the single screw extrusion process as well as on the physical, mechanical, and structural properties of the obtained biocomposite lignocellulosic extrudate with a low-density polyethylene matrix. 

The type of the tip used affects the distribution of filler particles, the effectiveness with which they are coated by the matrix material and the course of pore formation. A significant influence of these factors on water and moisture absorption and density was shown. From 40% bran content, the extrudate structure allows fluids to freely penetrate into the interior of the extrudate. The highest water and moisture absorption was observed in the samples prepared with the use of the cut rings mixing tip, which is associated with a higher volume of the working channel of this tip and the lower pressure of the material in relation to the other two tips, which enables pores to increase in volume.

The introduction of wheat bran to polyethylene caused a slight increase in sample stiffness and a significant decrease in tensile strength and elongation. The highest strength was obtained for samples extruded with the cut rings tip.

The thermal resistance of the extruded materials decreased in comparison to pure polyethylene, especially when using the cut rings tip. Oxidative decomposition of the samples did not lead to emissions of harmful organic gases. The addition of bran also reduced the degree of crystallinity of composites.

The color of the samples filled with bran changed to yellow and red tones with a simultaneous increase in brightness. This was related to the color and composition of the bran itself. The darkest and most yellow color was obtained for the cut rings tip, which is related to the filler distribution mechanism and the colored flakes being located closer to the surface of the extrudate. Although the cut rings tip, due to its geometry, employs distributive mixing, the quality of filler particle distribution is relatively poor, as confirmed by color tests.

Potential use of the tested composites will depend on the content of wheat bran. It is possible to use compositions with a high filling rate, i.e., from 40% by mass, for manufacturing products with a short life cycle. Such products usually do not have to meet strict durability requirements; they are disposed of after a short period of use. In such a case, the use of the cut rings tip would be justified. It allows for comparable mechanical strength, but at the same time also for the formation of pores of considerable size. The porous structure increases water and moisture absorption, accelerating degradation at the end of use. However, the studied compositions with wheat bran content below 40% by mass exhibited much lower water and moisture absorption levels, which means that they could be used for the production of parts with a longer life cycle or even periodically used outside. In this case, the use of pineapple and Maddock tips would be justified because of the much better homogenization and adhesion of filler particles to polyethylene due to less intense pore formation, which would potentially extend the life of the product.

## Figures and Tables

**Table 1:** Glass transition, melting, and crystallization temperatures and crystallinity degree of polyethylene and its composites obtained on the basis of DSC tests.

Sample	Heating I				Cooling	Heating II		
	T_m_ [°C]	ΔH_m_ [J/g]	X_c_ [%]	T_g_ [°C]	T_c_ [°C]	T_m_ [°C]	ΔH_m_ [J/g]	X_c_ [%]
PE	125.5	155.3	56.99	−113.6	108.3	125.0	177.0	64.96
Pineapple tip	126.0	-	-	−114.4	107.4	126.5	93.5	63.82
Cut rings tip	126.0	-	-	−111.7	107.7	125.7	87.6	59.80
Maddock tip	128.2	-	-	−106.6	107.1	127.0	80.4	54.88

**Table 2:** Results obtained from TG and DTG curves for PE, bran, and composites containing 50% wheat bran made using the Maddock, pineapple, and cut rings tips.

Sample	T_max1_ [°C]	Δ m_1_ [%]	T_max2_ [°C]	Δ m_2_ [%]	T_max3_ [°C]	Δ m_3_ [%]	T_max4_ [°C]	Δ m_4_ [%]	Rm [%]
PE	-	-	398	78.3	445	15.7	502	7.0	0
Bran	296	66.0	-	-	-	-	473	31.5	2.5
Pineapple tip	300	48.7	405	10.3	458	22.3	491	16.9	1.8
Cut rings tip	293	45.0	401	10.1	470	26.5	495	16.8	1.6
Maddock tip	299	32.3	409	29.6	458	19.4	495	17.4	1.3
